# Effect of the prosthetic index on stress distribution in Morse taper connection implant system and peri-implant bone: a 3D finite element analysis

**DOI:** 10.1186/s12903-022-02465-y

**Published:** 2022-09-30

**Authors:** Wen-tao Zhang, Kang-jie Cheng, Yun-feng Liu, Russell Wang, Yun-fang Chen, Yu-de Ding, Fan Yang, Lin-hong Wang

**Affiliations:** 1 Center for Plastic and Reconstructive Surgery, Department of Stomatology, Zhejiang Provincial People’s Hospital (Affiliated People’s Hospital, Hangzhou Medical College), No. 158 Shangtang Rd., Hangzhou, 310014 China; 2 College of Mechanical Engineering, Zhejiang University of Technology, Hangzhou, 310023 China; 3 Key Laboratory of Special Purpose Equipment and Advanced Processing Technology, Ministry of Education and Zhejiang Province, Zhejiang University of Technology, Hangzhou, 310023 China; 4 National International Joint Research Center of Special Purpose Equipment and Advanced Processing Technology, Zhejiang University of Technology, Hangzhou, 310023 China; 5 Department of Comprehensive Care, Case Western Reserve University School of Dental Medicine, Cleveland, OH 44106-4905 USA

**Keywords:** Implant–abutment connection, Platform switching, Platform matching, Morse taper, Prosthetic index, Finite element analysis

## Abstract

**Background:**

The combination of a prosthetic index with Morse taper connection was developed, with the purpose of making prosthetic procedures more precise. However, the presence of the index may compromise the mechanical performance of the abutment. The aim of this study is to evaluate the effect of prosthetic index on stress distribution in implant–abutment-screw system and peri-implant bone by using the 3D finite element methodology.

**Methods:**

Two commercial dental implant systems with different implant–abutment connections were used: the Morse taper connection with platform switching (MT-PS) implant system and the internal hex connection with platform matching (IH-PM) implant system. Meanwhile, there are two different designs of Morse taper connection abutment, namely, abutments with or without index. Consequently, three different models were developed and evaluated: (1) MT-PS indexed, (2) MT-PS non-indexed, and (3) IH-PM. These models were inserted into a bone block. Vertical and oblique forces of 100 N were applied to each abutment to simulate occlusal loadings.

**Results:**

For the MT-PS implant system, the maximum stress was always concentrated in the abutment neck under both vertical and oblique loading. Moreover, the maximum von Mises stress in the neck of the MT-PS abutment with index even exceed the yield strength of titanium alloy under the oblique loading. For the IH-PM implant system, however, the maximum stress was always located at the implant. Additionally, the MT-PS implant system has a significantly higher stress level in the abutment neck and a lower stress level around the peri-implant bone compared to the IH-PM implant system. The combined average maximum stress from vertical and oblique loads is 2.04 times higher in the MT-PS indexed model, and 1.82 times for the MT-PS non-indexed model than that of the IH-PM model.

**Conclusions:**

MT-PS with index will cause higher stress concentration on the abutment neck than that of without index, which is more prone to mechanical complications. Nevertheless, MT-PS decreases stress within cancellous bone and may contribute to limiting crestal bone resorption.

## Background

The concept of platform switching refers to the use of a narrower-diameter abutment placed on a larger-diameter implant platform, which results in movement of the implant–abutment junction inward toward the central axis of the implant and further away from the implant shoulder [1–3]. A number of clinical trials of platform-switched implants have reported that can significantly reduce crestal bone loss [4–9]. In addition, the platform-switched implants can provide extra surface area for the development and attachment of soft tissues, which is conducive to determining the biological width to produce excellent aesthetic results [10, 11]. Consequently, platform-switched implants have already been widely used clinically, especially for esthetic consideration in the anterior area [12].

Morse taper connections have been developed with the purpose of improving the interface between soft tissue and implant–abutment junction, and reducing the incidence of prosthetic complications [13, 14]. Furthermore, Morse taper connection implants with platform-switched abutments are associated with higher biomechanical stability and sealing capacity, as well as lower peri-implant marginal bone resorption [15–18].

However, it will be challenging for clinicians to guarantee the round-shaped abutment positioning on the Morse taper implant precisely. Thus, a prosthetic index was developed, aiming to combine the advantages of internal hex and Morse taper design, and thus facilitate prosthetic procedures [15, 16]. The prosthetic index is usually an internal hexagon or octagonal index, both inside the implant and incorporated with the abutment at the middle or bottom of conical contact area. Both the indexed and non-indexed abutments could be assembled to the indexed implant. Nevertheless, the presence of the index may compromise the biomechanical stability because it reduces the area of conical contact [19], and then the longevity of the implant system [16, 17, 19–21]. Significantly, a retrospective clinical study reported relatively frequent abutment fractures (2.2%) in Ankylos implant system, which is one kind of Morse taper connection with platform switching (MT-PS) implant system that incorporated index configuration both for the implant and abutment [22]. Unfortunately, the index factor was not considered in their study. However, a higher fracture rate of the MT-PS implant system with an indexed abutment was clinically observed in our retrospective study than in the non-indexed one [23]. Several experimental studies have been conducted to investigate the biomechanical performance of implant systems influenced by the presence of the index configuration. Yao et al. evaluated Morse taper implants with indexed and non-indexed abutments through in vitro fatigue test, the results noted that adding an internal index could provide an anti-rotational function, but at the same time, could compromise the anti-bending strength of the abutment [20]. Villarinho et al. investigated the effect of a positioning index on the abutment screw preload values of Morse taper connection implants, and it was concluded that indexed tapered abutments for single-crown restorations might represent greater biomechanical risk under function [24]. The influence of the prosthetic index on removal torque and tensile removal force of Morse taper connection abutments [16, 25], as well as bacterial microleakage of Morse taper implants [26], were evaluated through in vitro studies.

Apart from the index design, a screwless conical connection design has been demonstrated clinical success [27], however, lower mechanical resistance was observed when compared with the screw‐retained conical connection design [28]. Recently, the feasibility of abutment screw withdrawal after conical abutment settlement was evaluated to solve the difficult problems of screw loosening and screw fracture [29]. Although the conical implant–abutment connection system with index design passed the cyclic test, additional studies should be conducted to test the clinical feasibility [29]. Furthermore, Shash et al. reported that a novel one-piece implant structure could enhance the biomechanical stability of implant-bone system due to no implant–abutment–screw joint [30]; yet they are not commonly used for broad implants.

Considering the difficulty in performing in vivo studies, finite element analysis (FEA) has taken a major role in the study of the relationship between implant and bone [31, 32]. FEA provides the possibility to predict the stress distribution at implant assemblies and peri-implant bone [33]. A number of studies have been conducted using the FEA method mainly focused on stress distribution in bone and on the mechanics of implant and abutment connections [34–36]. Also, the resistance against rotation of a positioning hex in tapered internal connection implant systems was investigated using FEA [35]. Furthermore, Zancopé et al. evaluate the influence of the prosthetic index inside Morse taper implants on fracture resistance to implant due to the reduction of the titanium implant wall thickness [36].

However, to the best of the authors’ knowledge, there is no data in the literature demonstrating the mechanical characteristics of implant–abutment–screw system and the stress distribution influenced by the prosthetic index using FEA. Therefore, it is particularly necessary to establish an evidence-based scientific model to rationalize the clinical observations and experimental results. Thus, the objective of this study was to evaluate the effect of prosthetic index on stress distribution in implant–abutment–screw system and peri-implant bone by using the 3D finite element methodology. The null hypothesis was that the prosthetic index in Morse taper connection implant system would not negatively affected the mechanical stability of implant–abutment connection.

## Materials and methods

### Design and modeling of different implant systems

**Fig. 1:**
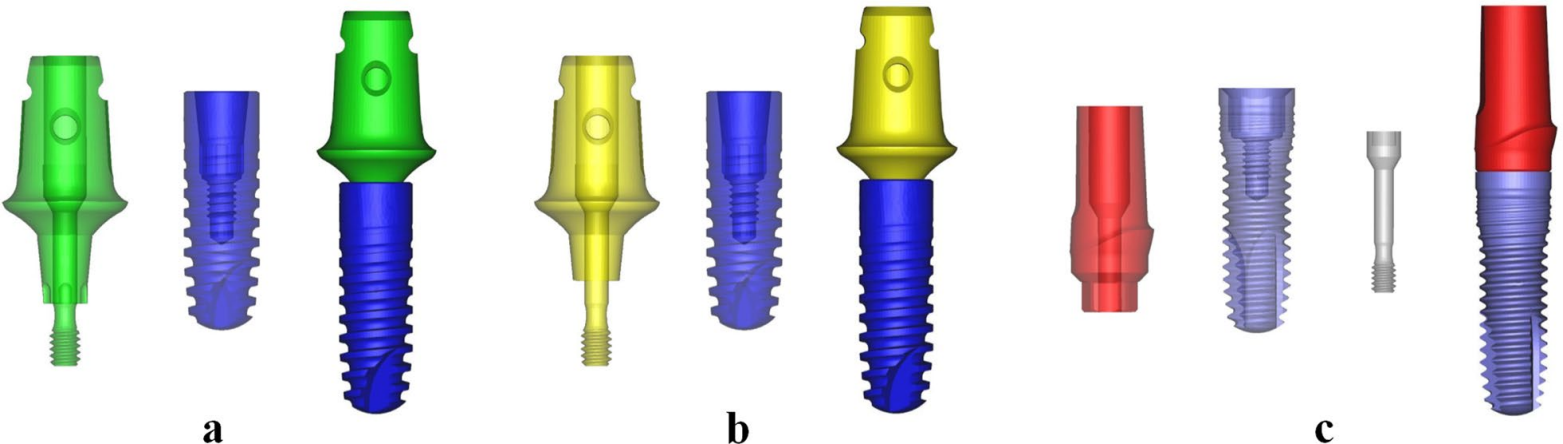
Computer-aided design geometry model of the three dental implant systems. **a** MT-PS implant system with an indexed abutment; **b** MT-PS implant system with a non-indexed abutment; **c** IH-PM implant system

The 3D geometrical models of the two dental implant systems (Fig. 1) with similar maximum diameters and lengths were created by copying the real implants using a 3D optical scanner (AutoScan-DS200 + ; Shining 3D Tech Co. Ltd., Hangzhou, China) and CAD software (NX 10.0; Siemens AG, Munich, Germany). Figure 1a shows the MT-PS implant system (Ankylos®, Dentsply Friadent GmbH, Mannheim, Germany) with an indexed component, whereas Fig. 1b has a non-indexed component. The two models are almost in the same geometry, but the indexed component is various in anti-rotational abutment compared to the non-indexed component. Figure 1c shows the IH-PM implant system (Bego® Implant Systems, Bremen, Germany). The detailed geometry and dimension of the two implant–abutment connections are shown in Table 1.

**Table 1:** Geometry and dimension of the two implant–abutment connections

Abutment design	Implant diameter × height (mm)	Gingival height (mm)	Abutment diameter (mm)	Abutment angle (°)
MT-PS indexed	3.5 × 11	1.5	3.84	0
MT-PS non-indexed	3.5 × 11	1.5	3.84	0
IH-PM	3.75 × 11.5	1–2	3.18	0

### Mandibular bone block model

**Fig. 2:**
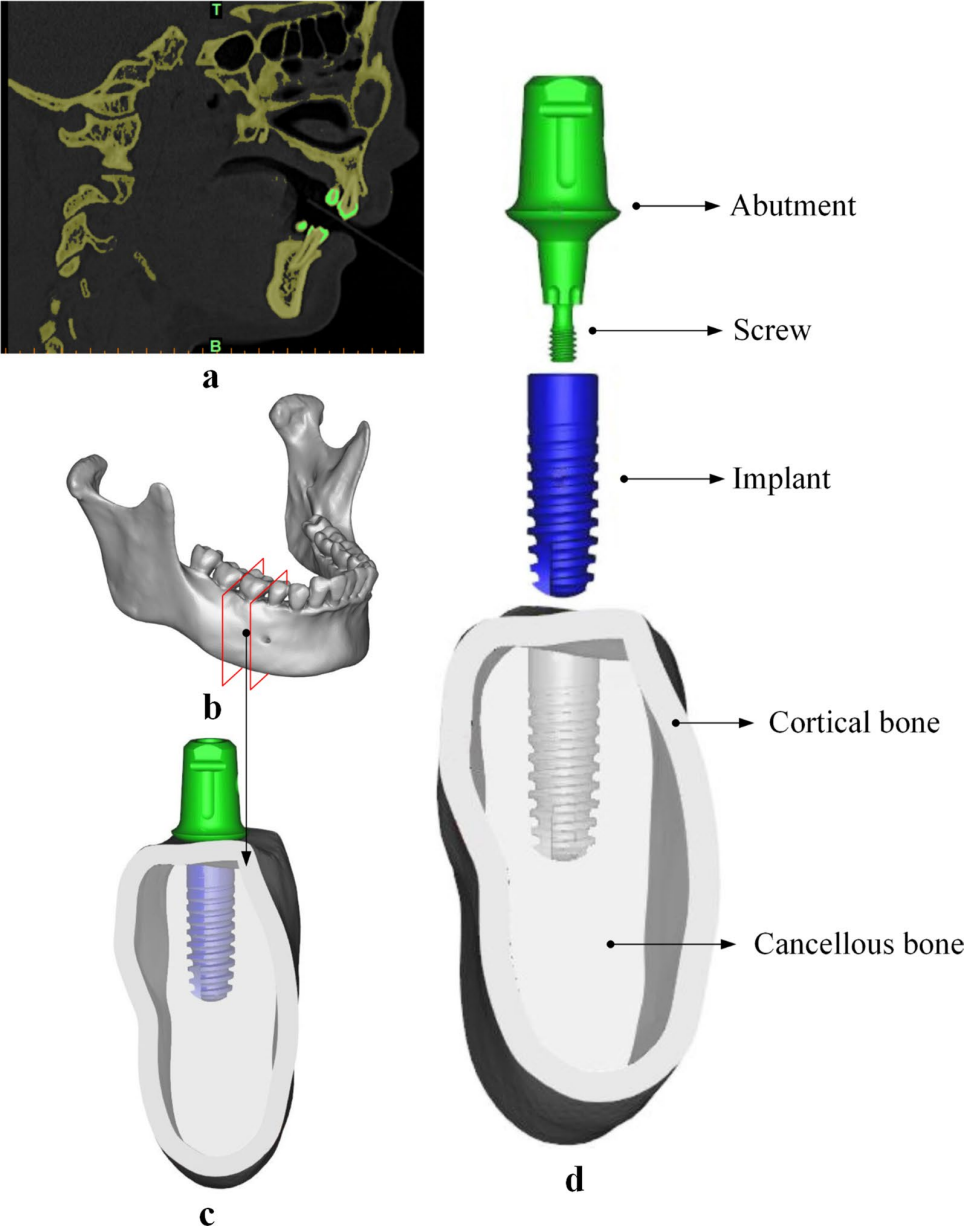
Assembly of mandibular bone block model and dental implant model. **a** One section from CBCT data; **b** The 3D mandible model was generated by Mimics; **c** MT-PS implant system with an indexed abutment was assembled with the mandibular bone model and abutment; **d** The models consisted of four parts: namely, abutment, implant, cortical bone and cancellous bone

CBCT images were obtained from a thirty years old healthy female volunteer with normal occlusion (Fig. 2a). The protocol for this study was approved by the Ethics Committee of Zhejiang Provincial People’s Hospital (Affiliated People’s Hospital, Hangzhou Medical College) (No. QT2022093). The mandibular bone block model was reconstructed based on the cross-sectional images of the right side first molar region (27 × 11 × 12 mm) using Mimics (V17.0, Materialise, Leuven, Belgium) software (Fig. 2b). The integration of mandibular bone block model and dental implant model was obtained by the Boolean operation functions using Magics (V20.03, Materialise, Leuven, Belgium) software (Fig. 2c). Assembly models of exploded view are shown in Fig. 2d.

### Material properties of assembly models

The abutment, screw, implant, cortical bone and cancellous bone were considered to be homogeneous, isotropic and linearly elastic materials [33, 37]. The Young’s modulus and Poisson’s ratio of the related materials used in the simulation of this study are shown in Table 2 [38, 39].

**Table 2:** Material properties used in the FE models

Materials	Young’s modulus (MPa)	Poisson’s ratios
Cortical bone	13,700	0.3
Cancellous bone	1370	0.3
Implant	112,000	0.33
Abutment	112,000	0.33
Screw	112,000	0.33

### Meshing

**Fig. 3:**
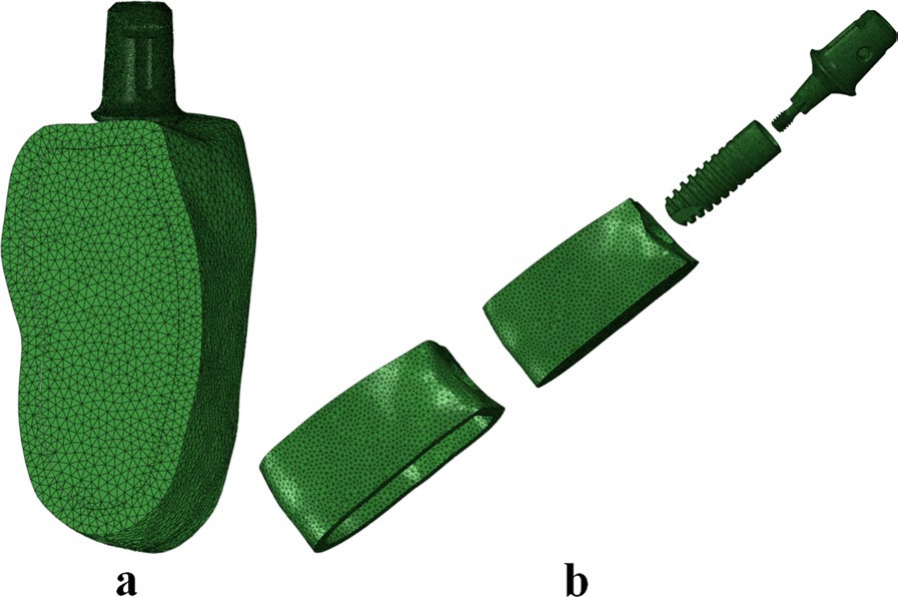
Finite element mesh models (take MT-PS implant system with an indexed abutment as an example). **a** Assembly model; **b** Part models

The assembly models were imported into Abaqus software (V6.13, Dassault Systèmes, Cedex, France) to generate tetrahedral meshes for subsequent simulations and calculations. The average element size of 0.5 mm was utilized as the meshing requirement for cortical bone and cancellous bone. A refined mesh (0.1 mm of average element size) was generated on the components of implant, abutment, screw, and the surrounding bone adjacent to the implant to guarantee proper geometrical and dimensional features (Fig. 3). The meshes were subject to the convergence test.

### Loads and boundary constraints

**Fig. 4:**
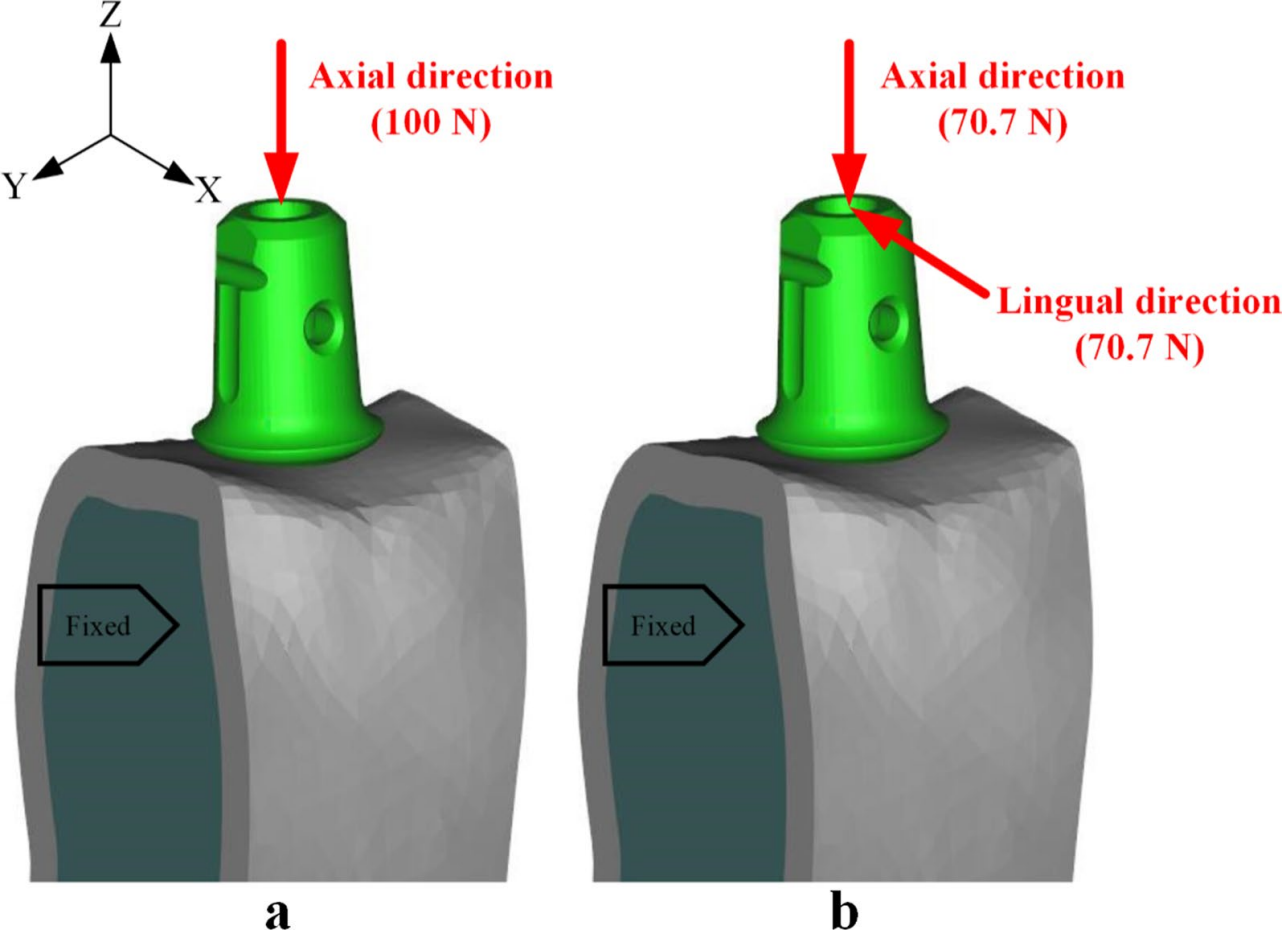
Loads and boundary constraints on the calculation model (take MT-PS implant system with an indexed abutment as an example). **a** The abutment was subjected to vertical load; **b** The abutment was subjected to oblique load

Same loads and boundary constraints were carried out for all calculation models (Fig. 4). Mesial and distal surfaces of cortical and cancellous bone were fixated in 6 degrees of freedom (Fig. 4, black). A vertical load (along the axis of implant) of 100 N or an oblique load (along the bucco-lingual direction, inclined at 45 degrees to the axis of implant) of 100 N was applied onto the occlusal surface of the abutment (Fig. 4, red) [37, 40, 41]. The 100 N value was measured by Kelly et al. [42] in the oral cavity. Biting forces on implants are similar to those reported for natural dentition [43]. Consequently, the 100 N vertical and oblique loads in this study were selected, which are conservative values for mean peak loads [43, 44]. During the process of setting the loadings, a reference point in the center of the abutment plane was first set; Subsequently, the coupling constraint between the coupling nodes of the abutment plane and the reference point was defined. Thus, the resultant loadings at the coupling nodes of the abutment plane are equivalent to the loadings at the reference point.

During the initial period of osseointegration, the peri-implant bone is imperfectly bonded to the implant surface. “Frictional” type was used to simulate the integration quality between peri-implant bone and the implant when it was placed immediately. The definition of frictional contact among abutment, screw and implant interface was dependent on the surface finishing (Table 3). The “frictional” coefficient was set as 0.4 and 0.5, respectively [45]. The rest of the contact surfaces were defined as ‘bonded’ type (Table 3) [46].

**Table 3:** Contact relationship

	Screw	Implant	Cortical bone	Cancellous bone
Abutment	Frictional	Frictional	–	–
Screw	–	Bonded	–	–
Implant	Bonded	–	Frictional	Frictional
Cortical bone	–	Frictional	–	Bonded

### Validation of calculation models

Validation of finite element models is extremely important as it is a solid basis for evaluating and improving reliable predictions of clinical treatment. However, there are unavoidable differences between the constructed physical model and finite element model in terms of the mechanical properties of mandibular bone block, the interaction between implant system and bone block. The geometry of the implant system components applied in this study was exactly the same as that of the clinical treatment. The mandibular bone block model was reconstructed from a human being. The material properties were assigned based on the previously published studies [38, 39]. The meshes were adequately refined until the relative errors of the maximum von Mises stress of the models were less than 1%. In the convergence models, the number of nodes and elements are shown in Table 4. The simulated loads boundary constraints were properly applied [37, 45, 46].

**Table 4:** Number of nodes and elements in the finite element models

Models	Number of nodes	Number of elements
MT-PS indexed	219,322	1,151,714
MT-PS non-indexed	215,275	1,131,389
IH-PM	225,001	1,171,056

## Results

**Fig. 5:**
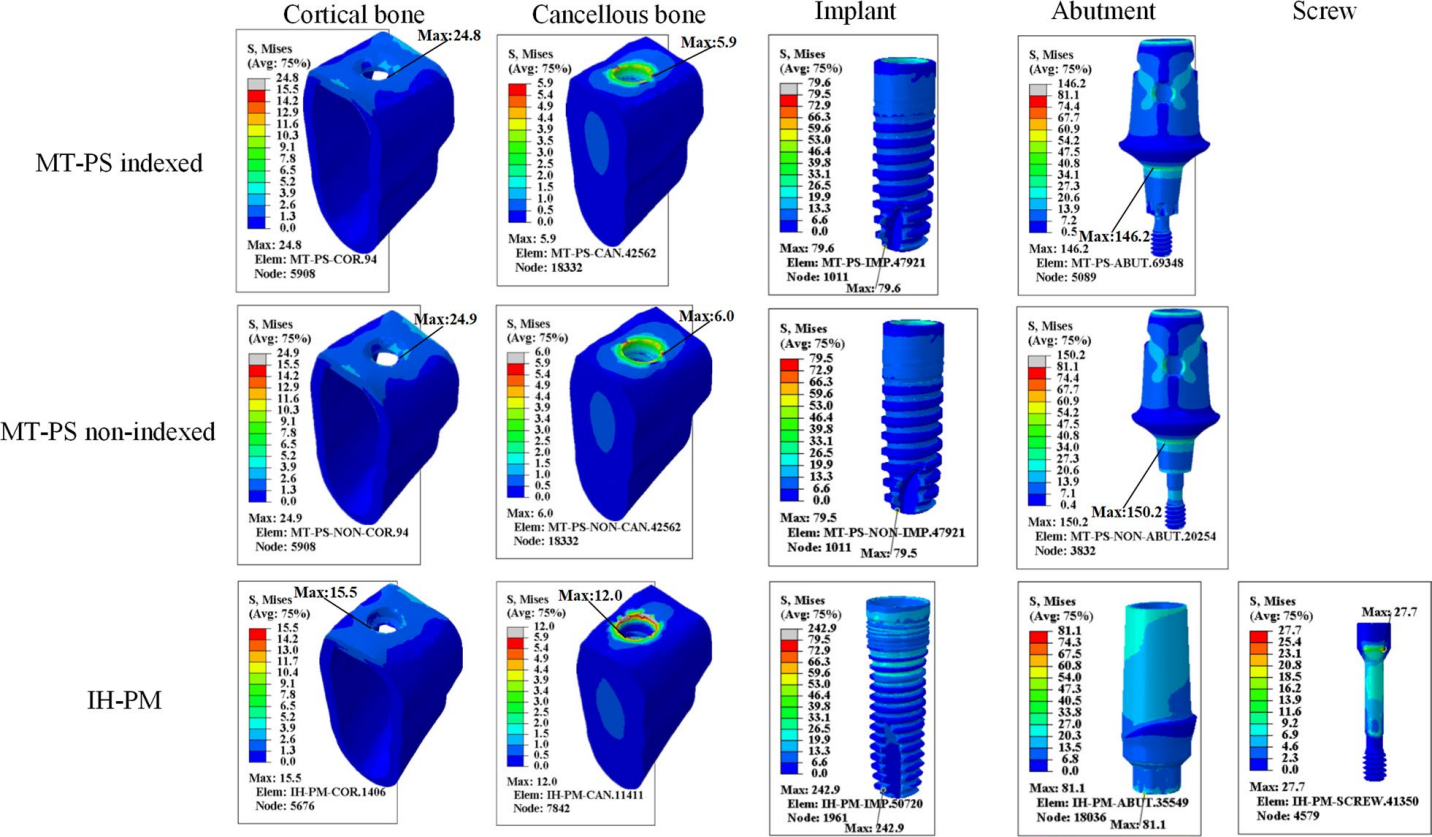
Von Mises stress distribution of all the components under vertical loading of 100 N

**Fig. 6:**
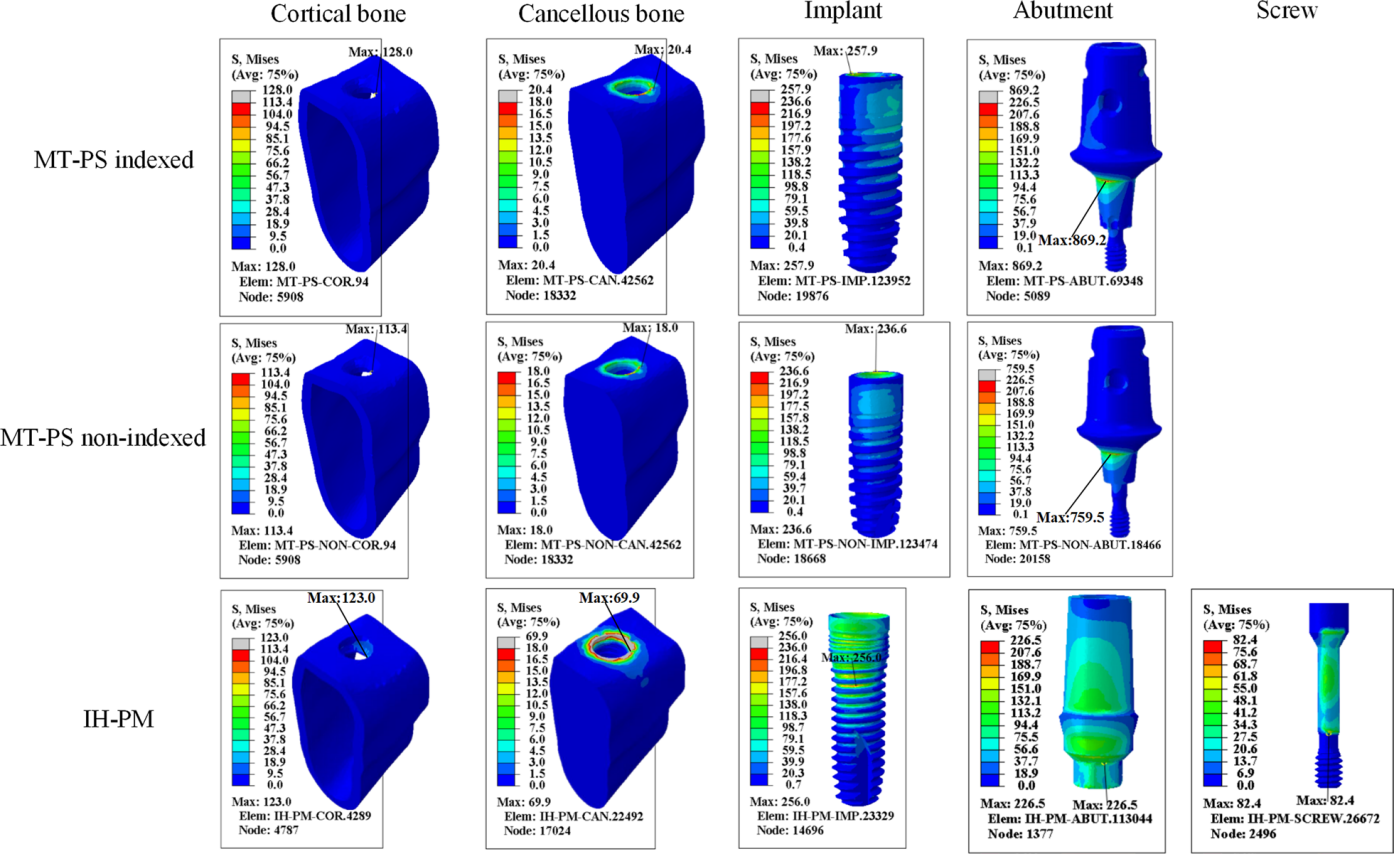
Von Mises stress distribution of all the components under oblique loading of 100 N

Figures 5 and 6 show von Mises stress distribution that occurred at the bone, implant, abutment and screw of three calculation models under vertical and oblique loading. For the MT-PS implant system, the maximum stress was always concentrated in the abutment neck under both vertical and oblique loading. Moreover, the maximum von Mises stress in the neck of the indexed MT-PS abutment was significantly higher than that of the non-indexed MT-PS abutment under the oblique loading. For the IH-PM implant system, however, the maximum stress was always located at the implant. Additionally, the MT-PS implant system has a significantly higher stress level in the abutment neck and a lower stress level around the peri-implant bone compared to the IH-PM implant system. The combined average maximum stress from vertical and oblique loads is 2.04 times higher in the MT-PS indexed model, and 1.82 times for the MT-PS non-indexed model than that of the IH-PM model.

Tables 5 and 6 summarize the results of maximum von Mises stress of all the components under vertical and oblique loading conditions.

**Table 5:** The maximum von Mises stress (MPa) of all the components under vertical loading

Components	MT-PS indexed	MT-PS non-indexed	IH-PM
Cortical bone	24.8	24.9	15.5
Cancellous bone	5.9	6.0	12.0
Implant	79.6	79.5	242.9
Abutment	146.2	150.2	81.1
Screw	NA	NA	27.7

**Table 6:** The maximum von Mises stress (MPa) of all the components under oblique loading

Components	MT-PS indexed	MT-PS non-indexed	IH-PM
Cortical bone	128.0	113.4	123.0
Cancellous bone	20.4	18.0	69.9
Implant	257.9	236.6	256.0
Abutment	869.2	759.5	226.5
Screw	NA	NA	82.4

## Discussion

The null hypothesis was rejected in view of the outcomes of this study. The prosthetic index in Morse taper connection implant system was negatively affected the mechanical stability of implant–abutment connection. Specifically, the performance of two different designs of MT-PS connection abutment were focused on, namely, indexed or non-indexed abutment. These implant–abutment–screw complexes were integrated into a human mandibular bone block. The mechanical effects of vertical and oblique loadings on the complex were investigated. The implant–abutment configurations that have been compared and analyzed in this study represent two commercially available dental implant systems, MT-PS implant system and IH-PM implant system.

Under vertical loading condition, the present study indicated that the maximum von Mises stress and stress distribution in MT-PS indexed model were extremely similar to those of MT-PS non-indexed model (Fig. 5; Table 5); However, the peri-implant bone (cancellous bone) of MT-PS implant has a smaller maximum stress (average 6.0 MPa) and more even stress distribution than IH-PM implant (12.0 MPa). It may imply that platform switching configuration could help to limit crestal bone resorption, which was consistent with the previous studies [47–50]. On the other hand, regarding the implant–abutment–screw system of MT-PS implant also shown a lower maximum stress (average 148.2 MPa) and more favorable stress distribution compared to those of IH-PM implant (242.9 MPa) [47, 51].

It should be kept in mind that it is mandatory to focus on oblique loading rather than vertical loading, which has been suggested to symbolize a realistic occlusal situation [33]. In all models and for both cortical and cancellous bone, the maximum von Mises stress increased dramatically under oblique loading compared to vertical loading. Furthermore, under oblique loading condition, the present study revealed that the cancellous bone stress significantly decreased if platform switching configuration was considered (approximately decreased 72.5%) (Fig. 6; Table 6). The overloading on peri-implant bone can lead to bone resorption, which has been reported [52]. Finally, it may result in loss of osseointegration [46] according to Frost’s Mechanostat Theory [53]. For the IH-PM model, the stress value of peri-implant bone (69.9 MPa) may exceed the yield strength of cancellous bone (50 MPa) [54], which may increase the risk of bone resorption and loss of osseointegration.

For the MT-PS model, we observed that the stress values around abutment neck, especially with an indexed component, were approached and even exceeded the yield strength of titanium alloy (780–950 MPa) [55]. These results are in agreement with Quaresma et al.’s study also using finite element methods [56], which indicated that greater von Mises stress was observed on the neck portion of the abutment-prosthesis complex in the conical implant. These results indicated that platform switching configuration decreased stress within peri-implant bone, as well as dramatically increased the stress on the abutment neck and screw. Hence, the present FEA study complemented the previous clinical study published by Shim & Yang [22], who reported relatively frequent abutment fracture incidence of 2.2% was observed, and all fractures occurred in the neck of the abutment and screw. Our documented follow-up data referring to implant complications showed similar results of the abutment neck fracture. In addition, a higher fracture rate of the MT-PS implant system with an indexed abutment was observed compared with the non-indexed one. However, the design of the fractured abutment, whether indexed or non-indexed, was not mentioned in the previous study [22].

In recent decade years, there have been few reports on the significant shortcomings by using platform switching configuration [22]. The results of the present study quantitatively revealed that the stress concentration on the abutment of MT-PS implant system can be a serious weakness that may lead to mechanical complications, including abutment and screw fracture, especially for the one with a prosthetic index. The reason for this phenomenon was due to the interruption of the stress flow, that is, the increase in the geometric structure difference between implant and abutment interface. In addition, the combination of an internal prosthetic index with Morse taper connection of MT-PS implant further reduces the micro-movement in the implant–abutment–screw system. In fact, numerous literatures have consistently reported the effectiveness of platform switching configuration in limit crestal bone resorption [4–9]. Similar conclusions were found in this study. After comparing the maximum stress values at bones for both MT-PS and IH-PM implant systems, the cancellous bone stress peaks were significantly reduced for the MT-PS implant system under oblique loading, suggesting decreased a risk of bone resorption and loss of osseointegration. Therefore, it was strongly recommended to adopt the platform switching configuration in the esthetic zones that were mainly subjected to oblique loading to maintain the soft and hard tissue as possible. But when the large oblique loading was unavoidable, especially in the non-esthetic zones, the platform switching configuration with an indexed abutment should be used cautiously.

The finite element model of this study has some limitations. All material properties in FEA were considered to be homogeneous and isotropic [33, 37], but this simplification was convenient to compare the simulation results. Furthermore, the dental crown was not modeled. The reason was to eliminate the confounding effect on the simulation results caused by the difficulty of accurately applying occlusal forces to the same position of the crown with complex shape. In addition, the stress distribution of the implant system was investigated without considering any individual factor. Through the FEA in this study, it was possible to improve comprehension of the detailed mechanical responses to the unexpected failure of the MT-PS implant system abutment, especially the indexed one. However, we suggested that the simulation results of this study should be promoted cautiously because FEA does not consider the bone remodeling process.

In view of the limitations of the present finite element model as well as the complexity of the biomechanics of mastication, future research, including both in vitro studies and clinical trials, will be carried out to validate the accuracy of the finite element model and clarify the mechanical mechanism of accidental failure of MT-PS implant system abutment.

## Conclusions

Based on the findings of FEA, it was suggested that MT-PS with index will cause higher stress concentration on the abutment neck than that of without index, and even exceeded the yield strength of titanium alloy, which is more prone to mechanical complications. Nevertheless, MT-PS decreases stress within cancellous bone and may contribute to limiting crestal bone resorption. This work provided computational modeling reference for clinicians to select a suitable implant–abutment connection system for different clinical situations as well as dental implant researchers to optimize the design of dental implants with consideration of the limitations of this FEA.

## Data Availability

All data are calculated by the software itself. The datasets used and/or analysed during the current study available from the corresponding author on reasonable request.
